# Elements of Geology for Popular Use; Containing a Description of the Geological Formations and Mineral Resources of the United States

**Published:** 1841-01

**Authors:** 


Art. VIII.—*Elements of Geology for popular Use; containing a Description of the Geological Formations and Mineral Resources of the United States.* By Charles A. Lee, m.d.—*New York*, 1840. 12mo, pp. 375.

This is another of those excellent manuals for which America is indebted to Dr. Lee, and which successfully aim at bringing down the lofty truths of science to popular apprehension. The present little volume cannot fail to be most useful to the class for whom it is written, and will add to its author's reputation. We do not know a better work for putting into the hands of beginners in geology, and we therefore notice it in our pages. It has the usual American blemish, of wretched woodcuts; but for this the author is not responsible.

